# Correction: Porous monoliths synthesized *via* polymerization of styrene and divinyl benzene in nonaqueous deep-eutectic solvent-based HIPEs

**DOI:** 10.1039/c8ra90056f

**Published:** 2018-06-29

**Authors:** M. G. Pérez-García, A. Carranza, J. E. Puig, J. A. Pojman, F. del Monte, G. Luna-Bárcenas, J. D. Mota-Morales

**Affiliations:** Instituto de Ciencia de Materiales de Madrid-ICMM, Consejo Superior de Investigaciones Científicas-CSIC Campus Cantoblanco 28049 Madrid Spain delmonte@icmm.csic.es; Department of Chemistry, Louisiana State University Baton Rouge Louisiana 70803 USA; Ingeniería Química, Universidad de Guadalajara Guadalajara Jalisco 44430 Mexico; Centro de Investigación y de Estudios Avanzados Unidad Querétaro Querétaro 76230 Mexico gluna@qro.cinvestav.mx; Cátedras Conacyt at Centro de Nanociencias y Nanotecnología-UNAM Ensenada Baja California 22860 Mexico mota_josue@hotmail.com

## Abstract

Correction for ‘Porous monoliths synthesized *via* polymerization of styrene and divinyl benzene in nonaqueous deep-eutectic solvent-based HIPEs’ by M. G. Pérez-García *et al.*, *RSC Adv.*, 2015, **5**, 23255–23260.

The authors regret that there was an error in the results and discussion section of the original article. On page 23257, the text read, “The surfactant employed here was sorbitan monooleate”. This should have read, “The surfactant employed here was sorbitan stearate”.

The Royal Society of Chemistry apologises for these errors and any consequent inconvenience to authors and readers.

## Supplementary Material

